# Edge–Point Cloud Fusion for Geometric Fitting of Cylinder Parameters Using Single-View RGB-D Data

**DOI:** 10.3390/s26051687

**Published:** 2026-03-07

**Authors:** Huayan Zhang, Jiaxin Liu, Zhongkui Wang

**Affiliations:** Department of Robotics, Ritsumeikan University, 1-1-1 Nojihigashi, Kusatsu 525-8577, Shiga, Japan; gr0607pk@ed.ritsumei.ac.jp (H.Z.); gr0500ip@ed.ritsumei.ac.jp (J.L.)

**Keywords:** geometric fitting, cylinder parameters, edge–point cloud fusion, RGB-D data, parameter optimization

## Abstract

Cylinders are common in both industrial and daily settings. Accurate geometric fitting of their parameters, including position, orientation, and radius, is important in real-world perception tasks and industrial applications. At present, consumer-level RGB-D cameras provide three-dimensional (3D) point cloud data with acceptable accuracy and are widely adopted in various sensing applications. Consequently, this task is typically formulated as a geometric fitting problem based on point cloud data. However, point cloud data acquired from such sensors often contain noise, particularly when scanning curved surfaces, which directly degrades the performance of point cloud-based fitting methods. In this paper, we propose an edge–point cloud fusion approach for the geometric fitting of cylinder parameters from single-view RGB-D data. Our approach leverages two-dimensional (2D) image-domain edge constraints together with point cloud data, then fuses them in a unified formulation to jointly optimize cylinder parameters. By explicitly incorporating reliable edge information, our method effectively mitigates the effects of noise in point cloud data. We evaluate the proposed method using real-world RGB-D data, and the experimental results show that our approach achieves significant improvements in both accuracy and robustness.

## 1. Introduction

Cylinders are fundamental geometric primitives that are widely adopted in both industrial (pipes, tanks, etc.) and real-world (cans, bottles, etc.) environments. Accurate estimation of their parameters (including orientation, position and radius) is important for industrial applications and perception tasks. This capability ensures the reliable reconstruction of cylindrical structures, such as modeling pipeline plants [1] and Building Information Modeling (BIM) [2,3], while also serving as a basis for robotic manipulation [4,5,6]. Point cloud data provide 3D geometry information and represent a primary data source for such applications. In this context, the estimation of cylinder parameters is typically formulated as a geometric fitting task based on point cloud data, making it a fundamental problem in geometric modeling.

Traditional methods have been widely adopted in prior research works, which mainly include Random Sample Consensus (RANSAC) [7], the Hough transform [8,9], Principal Component Analysis (PCA) [10,11], and least squares-based fitting methods [12,13,14,15]. While these methods achieve good performance on well-conditioned point cloud data, maintaining accuracy on complex real-world data remains challenging. This becomes more noticeable in practical applications where point cloud data are acquired by consumer-level 3D scanners such as RGB-D cameras. Due to their low cost and acceptable accuracy, these types of sensors have become widely adopted; however, point cloud data from such sensors typically contain noise [16,17], particularly for complex curved surfaces. This severely affects the performance of traditional methods, leading to axial drift, unreliable radius estimation, and inaccurate model fitting.

Since traditional methods are sensitive under such sensing conditions, researchers have made efforts to extend the classical framework. These extensions mainly include integrating the curvature information into the voting process [9], improving the normal estimation [18], suppressing unreliable depth measurements near object boundaries [1], and clustering-based filtering strategies [19]. These methods continue to follow multi-stage frameworks in which the central axis and the radius are estimated in a step-by-step scheme. This design tends to produce errors early on, which are then transferred and accumulated in later stages. Consequently, the upper bound in terms of overall performance is restricted. To this end, Zhang et al. [15] proposed a least squares-based optimization framework that can jointly optimize cylinder parameters; although this method achieves better fitting to input point cloud data, parameter estimation accuracy is still influenced by the complex noise in real-world data.

While depth measurements are susceptible to noise, color images from RGB-D cameras contain complementary geometric information. In view of this observation, Kawagoshi et al. [20] attempted to utilize edge cues other than point clouds alone for the cylinder fitting. However, their work focuses on radius estimation and relies solely on a viewpoint-specific modeling assumption, making this method less generalizable. Therefore, it is essential to investigate more effective methods for integrating multimodal geometric information to fit cylinder parameters in real-world sensing scenarios.

In this paper, we propose an optimization-based geometric fitting method for estimating cylinder parameters from single-view RGB-D observations. Our method is designed as a backend geometric refinement module, assuming that cylindrical regions are available from upstream pipelines and focusing on reducing the effect of noisy point cloud data on parameter estimation. Unlike prior work [20], our formulation explicitly considers edge alignment constraints that are inherent from the projected cylinder geometry. We introduce a complementary modality fusion strategy that combines 3D point measurements with image-domain edge information within a unified optimization framework, allowing reliable edge information to compensate for deviations in the estimated parameters. To assess the effectiveness of the proposed method, we evaluate it on real-world RGB-D data under controlled settings. The experimental results show that our approach achieves significant improvements in both accuracy and robustness. The contributions of this paper are as follows:

- We propose an edge–point cloud fusion method for geometric fitting of cylinder parameters.
- We present a unified fusion formulation and an optimization procedure to jointly estimate all cylinder parameters under constraints derived from both point measurements and edge observations.
- We validate the effectiveness of our approach and demonstrate significant performance improvements on real-world RGB-D data.

The remainder of this paper is organized as follows: Section 2 and Section 3 provide the notations, camera model, and geometric formulations of the cylinder; Section 4 details the proposed edge–point cloud fusion method for cylinder parameter estimation; Section 5 provides comprehensive experiments on robotic acquisition datasets and real-world pipe scenarios; Section 6 discusses the proposed method; finally, Section 7 concludes the paper and outlines future work.

## 2. Notations and Preliminaries

### 2.1. Notations

We use lowercase and uppercase letters for scalars (e.g., a∈R), bold lowercase letters for column vectors (e.g., $a\in R^{n}$), and bold uppercase letters for matrices (e.g., $A\in R^{m\times n}$). For a vector a, its *i*-th entry is denoted by $a_{i}$. For a matrix A, $A_{ij}$ denotes its *i*-th row and *j*-th column scalar, while $a_{i}$ denotes its *i*-th column vector. The operator ${\left(\cdot \right)}^{⊤}$ denotes the transpose, ${\left(\cdot \right)}^{-1}$ denotes the inverse, and ∥·∥ denotes the $\ell_{2}$ norm. The $\ell_{2}$-normalized vector is defined as $\hat{a}=a/∥a∥$. The operator $\tilde{\left(\cdot \right)}$ converts a vector into its homogeneous coordinate representation, e.g., $\tilde{a}={\left[a^{⊤},1\right]}^{⊤}$. The identity matrix of size n×n is $I_{n}$. The zero matrix of size m×n is $0_{m\times n}$. The 2D and 3D rotation groups are denoted by SO(2) and SO(3), respectively. The capitalized exponential map Exp(·) maps a vector element to its corresponding element in the group space [21]. For SO(2) and SO(3) groups, they are defined as follows:

(1) $$\begin{array}{cc} \; & \mathrm{Exp}\left(\phi \right)=\left[\begin{array}{cc} \cos\phi & -\sin\phi \\ \sin\phi & \cos\phi \end{array}\right],\\ \; & \mathrm{Exp}\left(\theta \right)=I_{3}+\sin\left(∥\theta ∥\right){\left[\hat{\theta }\right]}_{\times }+\left(1-\cos\left(∥\theta ∥\right)\right){\left[\hat{\theta }\right]}_{\times }^{2}, \end{array}$$

where ϕ∈R, $\theta \in R^{3}$, and ${\left[\cdot \right]}_{\times }$ denotes the skew-symmetric operator applied to a 3D vector θ:

(2) $${\left[\theta \right]}_{\times }=\left[\begin{array}{ccc} 0 & -\theta_{3} & \theta_{2}\\ \theta_{3} & 0 & -\theta_{1}\\ -\theta_{2} & \theta_{1} & 0 \end{array}\right].$$

Finally, we denote the depth image by D:Ω→R, where $\Omega \subset R^{2}$ represents the image domain.

### 2.2. Camera Model

As the point cloud data used for cylinder fitting are reconstructed from depth images, we briefly review the camera model. We assume a well-calibrated RGB-D camera such that the corresponding color and depth images are registered and rectified. Given a pixel coordinate $u\in R^{2}$ and its depth measurement D(u)∈R, the 3D point $p\in R^{3}$ is recovered via the inverse projection mapping under the standard pinhole camera model [22]:

(3) $$p=D\left(u\right)K^{-1}\tilde{u},$$

where K denotes the camera intrinsic matrix:

(4) $$K=\left[\begin{array}{ccc} f_{x} & 0 & c_{x}\\ 0 & f_{y} & c_{y}\\ 0 & 0 & 1 \end{array}\right],$$

where $f_{x},f_{y}$ are the focal lengths and $c_{x},c_{y}$ represent the coordinates of the principal point.

## 3. Geometric Formulation of the Cylinder

### 3.1. Parametric Representation of the Cylinder

**Figure 1:**
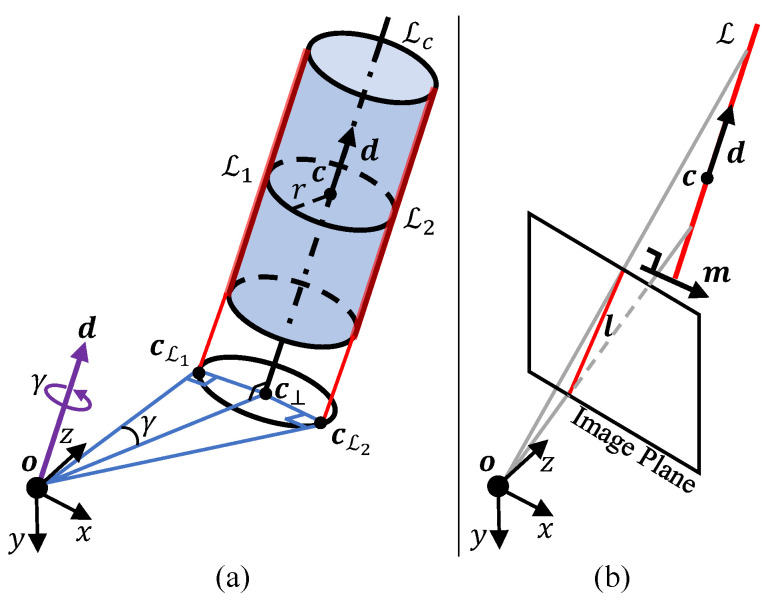
Cylinder geometry and projection model: (**a**) parametric representation of a cylinder with central axis $L_{c}$ and radius *r*, where $L_{1}$ and $L_{2}$ denote two visible longitudinal edges in the viewing direction; (**b**) projection of a 3D line L onto the image plane, where l is the projected 2D line and m is the moment vector.

An infinite 3D cylinder has five degrees of freedom (DoFs): four DoFs for the central axis $L_{c}$ and one for the radius r∈R, as shown in Figure 1a. In this paper, we adopt three parameterizations of the central axis: the point-direction form and Plücker line coordinates for geometric computation, and the orthonormal representation for optimization.

#### 3.1.1. Point-Direction Form

Geometrically, the central axis $L_{c}$ is a 3D line. It can be represented by the point-direction form {c,d}, where $c\in R^{3}$ is a point on the central axis and $d\in R^{3}$ denotes its direction. However, this representation uses six parameters for a 3D line with four DoFs, resulting in redundancy and potential numerical instability for optimization. To address this issue, we introduce the orthonormal representation [23], which provides a compact and well-conditioned parameterization for the optimization problem.

#### 3.1.2. Plücker Line Coordinates

Since the orthonormal representation is derived from the Plücker line coordinates [24], we first review this concept. The Plücker line coordinates are defined as $\left\{m,\hat{d}\right\}$, where m is the moment vector given by

(5) $$m=c\times \hat{d},$$

which is perpendicular to the interpretation plane containing the central axis and the origin. Here, $\hat{d}$ denotes the normalized direction vector. Due to the existence of Plücker constraints $m^{⊤}\hat{d}=0$ and $∥\hat{d}∥=1$, Plücker line coordinates have four DoFs in total, and as such provide a compact representation.

#### 3.1.3. Orthonormal Representation

To eliminate the Plücker constraints and enable unconstrained optimization, the orthonormal representation [23] is introduced. This representation parameterizes a 3D line using a pair (U,W)∈SO(3)×SO(2), which is derived from the Plücker line coordinates:

(6) $$\left\{\begin{array}{cc} U & =\left[\begin{array}{ccc} \hat{m} & \hat{d} & \frac{m\times \hat{d}}{∥m\times \hat{d}∥} \end{array}\right]\in \mathrm{SO}\left(3\right),\\ W & =\frac{1}{\sqrt{{∥m∥}^{2}+1}}\left[\begin{array}{cc} ∥m∥ & -1\\ 1 & ∥m∥ \end{array}\right]\in \mathrm{SO}\left(2\right). \end{array}\right.$$

This results in a minimal four-DoF representation of a 3D line without Plücker constraints. This representation, serving as the parameterization of the central axis $L_{c}$, enables joint optimization of all cylinder parameters on manifolds [15].

#### 3.1.4. Representation Conversion

The conversion from orthonormal representation to Plücker line coordinates is given by

(7) $$\left\{\begin{array}{cc} m & =\frac{W_{11}}{W_{21}}\;u_{1},\\ \hat{d} & =u_{2}. \end{array}\right.$$

Once $\left\{m,\hat{d}\right\}$ are obtained, the point on the 3D line closest to the origin is computed as

(8) $$c_{⊥}=\hat{d}\times m,$$

yielding the point-direction form. This establishes the correspondence among the three parameterizations considered in this study.

### 3.2. Projection of the Cylinder onto the Image Plane

The projection of a cylinder onto the image plane is represented by two visible edges [25]. We first recall how to project a 3D line onto the image plane, then derive the projection of the cylinder’s edges.

#### 3.2.1. Projection of a 3D Line

A 3D line represented in Plücker line coordinates $\left\{m,\hat{d}\right\}$ projects onto the image plane using the mapping $\pi_{L}\left(\cdot \right)$ [26], defined as follows:

(9) $$l=\pi_{L}\left(m\right)=K_{L}m,$$

where $l\in R^{3}$ denotes the homogeneous representation of the 2D line in the image plane, as shown in Figure 1b. The line projection matrix $K_{L}$ is determined by the camera’s intrinsic parameters

(10) $$K_{L}=\left[\begin{array}{ccc} f_{y} & 0 & 0\\ 0 & f_{x} & 0\\ -f_{y}c_{x} & -f_{x}c_{y} & f_{x}f_{y} \end{array}\right].$$

#### 3.2.2. Projection of Cylinder Edges

Given the central axis $L_{c}$ of a cylinder, the 3D visible edges $L_{j}$ (j=1,2) along with their 2D projections $l_{j}$ are derived through geometric computation. As shown in Figure 1b, $c_{⊥}$ and $c_{L_{j}}$ denote the points on the corresponding 3D lines that are closest to the camera optical center o. The angle γ is defined as the angle between the vectors from o to $c_{⊥}$ and $c_{L_{j}}$. According to the trigonometric relationship within the right triangle formed by the hypotenuse $∥c_{⊥}∥$ and the opposite side *r*, the angle γ is computed as follows:

(11) $$\gamma =\sin^{-1}\frac{r}{∥c_{⊥}∥}.$$

Once the angle γ is determined, $c_{L_{j}}$ are obtained by rotating $c_{⊥}$ about the axis through o with direction d by rotation angles of ±γ coupled with a scaling operation. The closed-form expression is given by

(12) $$\begin{array}{cc} c_{L_{j}} & =\cos\gamma \mathrm{Exp}\;\left({\left(-1\right)}^{j-1}\gamma {\left[\hat{d}\right]}_{\times }\right)c_{⊥},\;j=1,2. \end{array}$$

With $c_{L_{j}}$ established and the direction vector d inherited from $L_{c}$, the 3D visible edges $L_{j}$ are fully specified. The moment vectors $m_{L_{j}}$ are determined using Equation (5), and their projections are subsequently obtained via the mapping defined in (9):

(13) $$l_{j}=\pi_{L}\left(m_{L_{j}}\right),\;j=1,2.$$

To simplify notation, we denote this complete process as

(14) $$\left\{l_{1},l_{2}\right\}=\pi_{c}\left(L_{c},r\right).$$

## 4. Geometric Fitting of Cylinder Parameters via Edge–Point Cloud Fusion

### 4.1. Problem Formulation

We assume that a segmentation mask M corresponding to the visible cylindrical surface of the target object is provided in the RGB image, and that its two longitudinal edges are available. We denote the observed edge segments as the set $Q_{\mathrm{obs}}=\left\{O_{1},O_{2}\right\}$, where each $O_{m}=\left\{q_{m}^{1},q_{m}^{2}\right\}\subset R^{2}$ represents an edge segment specified by its two endpoints in pixel coordinates. The point cloud data from the cylindrical surface are obtained by back-projecting the masked depth image, and are denoted as $P={\left\{p_{i}\in R^{3}\right\}}_{i=1}^{N}$.

**Figure 2:**
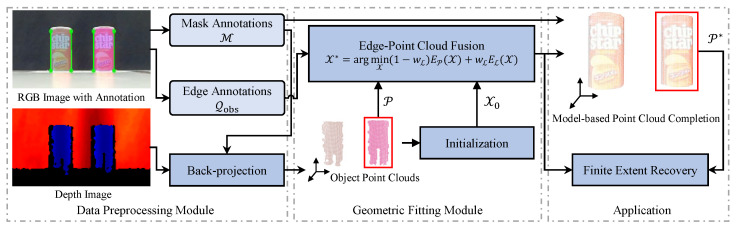
Flowchart of the proposed approach. Given annotated RGB-D data, a weighted edge–point cloud fusion approach is proposed to jointly exploit edge observations and point cloud data for geometric fitting of cylinder parameters. The estimated parameter $X^{*}$ enables model-based point cloud completion and finite-extent recovery.

An overview of the proposed method is shown in Figure 2. We take the preprocessed data as input into the geometric fitting module, which integrates point cloud data P and edge observations $Q_{\mathrm{obs}}$ to jointly optimize the cylinder parameters. The objective function is formulated as a weighted combination of two energy terms:

(15) $$F\left(X\right)=\left(1-w_{L}\right)E_{P}\left(X\right)+w_{L}E_{L}\left(X\right),$$

where X={U,W,r} represents the complete set of cylinder parameters using an orthonormal representation [15] that models the target as a visible segment of an infinite cylinder, enabling parameter estimation from observed cylindrical surface fragments without requiring end-face visibility. The objective function consists of two key components:

1. The point-to-cylinder energy term $E_{P}\left(X\right)$ ensures 3D geometric consistency, optimizing the model parameters X against observed point cloud data P.
2. The edge alignment energy term $E_{L}\left(X\right)$ constrains the pair of projected cylinder edges derived from X to align with the 2D edge annotations $Q_{\mathrm{obs}}$, ensuring spatial–visual consistency.

The edge fusion weight $w_{L}$ balances the contributions of the two energy terms. In the following sections, we detail the formulation of the energy terms and the optimization strategy. The estimated cylinder parameters provide a compact geometric representation of the target object, enabling geometric processing beyond parameter estimation. As an application, we show that the estimated model parameters X enable recovery of the cylinder centroid and length as well as model-based completion of the point cloud.

### 4.2. Point-to-Cylinder Energy Term

This term quantifies the geometric deviation of the observed points P from the estimated cylinder surface. We define the point-wise error term $e_{i}\left(X\right)$ as the signed minimal distance from a point $p_{i}$ to the cylinder surface:

(16) $$e_{i}\left(X\right)=∥m\left(U,W\right)-p_{i}\times \hat{d}\left(U\right)∥-r.$$

Consequently, the total point measurement energy $E_{P}\left(X\right)$ is computed as the mean squared error over all *N* observations:

(17) $$E_{P}\left(X\right)=\frac{1}{N}\sum_{i=1}^{N}e_{i}^{2}\left(X\right).$$

### 4.3. Edge Alignment Energy Term

To avoid scale ambiguities and maintain metric consistency with $E_{P}$, we define the edge alignment term in 3D space, allowing the weight $w_{L}$ to directly control the relative contributions of the two terms. Although the point cloud data corresponding to the annotation set $Q_{\mathrm{obs}}$ can be reconstructed from the depth image for error formulation, the resulting measurements are frequently missing or unreliable in regions near object boundaries. For this reason, we develop a geometry-based approach that back-projects the annotated endpoints by intersecting their viewing rays with a plane derived from the model parameters X. Notably, this approach does not rely on depth measurements at edge pixel locations. Therefore, even when depth data are missing near boundaries, the edge alignment term remains well-defined and provides effective geometric constraints. We first describe the data association strategy used to establish correspondences between the model parameters X and the observed edge annotations $Q_{\mathrm{obs}}$, then present the formal definition of the edge alignment energy.

#### 4.3.1. Edge-to-Model Data Association

To establish correspondences for the observed edge annotations $Q_{\mathrm{obs}}$, we generate the projected edges $\left\{l_{1},l_{2}\right\}$ from the cylinder parameters X using (14). Let $Q=\{Q_{l_{1}},Q_{l_{2}}\}$ denote the set of partitioned observations, where $Q_{l_{j}}=\left\{q_{l_{j}}^{1},q_{l_{j}}^{2}\right\}\subset R^{2}$ represents the specific segment in $Q_{\mathrm{obs}}$ assigned to the projected line $l_{j}$. The optimal association is determined by minimizing the cumulative alignment error. We construct a cost matrix $D\in R^{2\times 2}$, where each entry $D_{mj}$ quantifies the geometric distance between the *m*-th observed segment $O_{m}$ and the *j*-th projected line $l_{j}$:

(18) $$D_{mj}=\sum_{k=1}^{2}d\left(q_{m}^{k},l_{j}\right),\;m,j\in \left\{1,2\right\},$$

where d(q,l) denotes the perpendicular distance from an endpoint q to a line l:

$$d\left(q,l\right)=\frac{|l^{⊤}\tilde{q}|}{\sqrt{l_{1}^{2}+l_{2}^{2}}}.$$

The assignment is obtained by comparing the cost of the direct correspondence (represented by the diagonal sum $D_{11}+D_{22}$) against the swapped correspondence (represented by the anti-diagonal sum $D_{12}+D_{21}$). Accordingly, the matched segments $\left\{Q_{l_{1}},Q_{l_{2}}\right\}$ are determined by

(19) $$\begin{array}{cc} Q_{l_{1}} & =\left\{\begin{array}{cc} O_{1}, & D_{11}+D_{22}\leq D_{12}+D_{21},\\ O_{2}, & \mathrm{otherwise}, \end{array}\right.\\ Q_{l_{2}} & =\left\{\begin{array}{cc} O_{2}, & D_{11}+D_{22}\leq D_{12}+D_{21},\\ O_{1}, & \mathrm{otherwise}. \end{array}\right. \end{array}$$

The procedure for establishing these correspondences is detailed in Algorithm 1. Note that because the projected lines $\left\{l_{1},l_{2}\right\}$ are implicitly dependent on the model parameters X, the edge correspondence process is updated throughout the optimization process.


**Algorithm 1:** Data Association for Edge Alignment


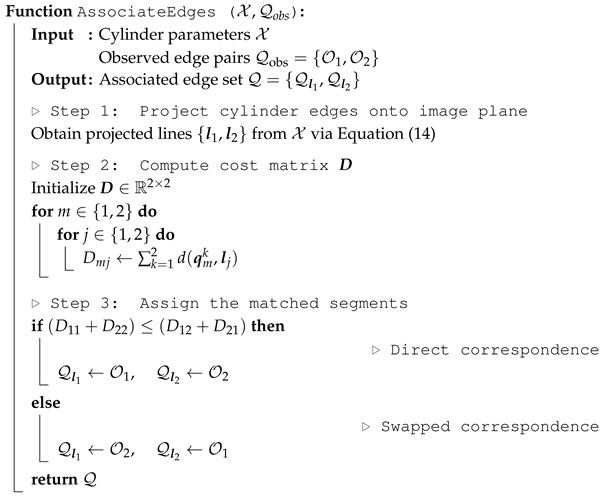





#### 4.3.2. Energy Term Formulation

**Figure 3:**
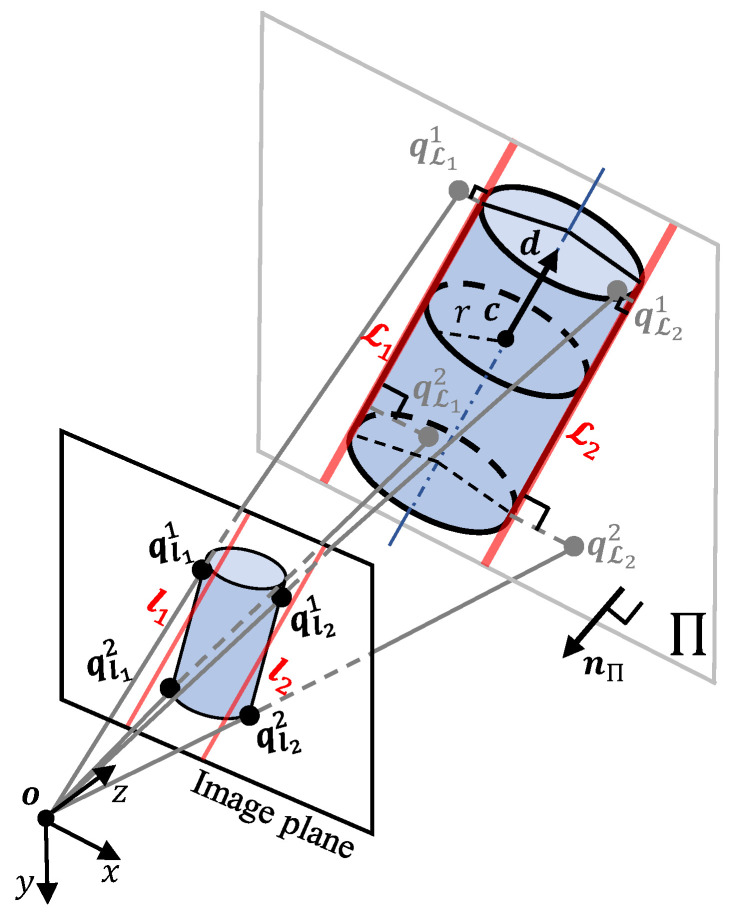
Back-projection geometry for the edge alignment energy term. The image endpoint $q_{l_{j}}^{k}$ defines a viewing ray emitted from the optical center o. The visible edges $L_{1}$ and $L_{2}$ derived from the cylinder parameters X define a reference plane Π. The back-projected 3D point $q_{L_{j}}^{k}$ is obtained as the intersection of the viewing ray with plane Π.

With the established correspondences, the edge alignment energy $E_{L}\left(X\right)$ is formulated through a geometry-based back-projection strategy. As illustrated in Figure 3, each endpoint $q_{l_{j}}^{k}\in Q$ defines a viewing ray $v_{l_{j}}^{k}$ originating from the camera optical center o:

(20) $$v_{l_{j}}^{k}=K^{-1}{\tilde{q}}_{l_{j}}^{k}.$$

A reference plane Π is constructed from the cylinder parameters X, and is spanned by the two visible edges:

(21) $$\left\{\begin{array}{cc} n_{\Pi }\left(X\right) & =\left(c_{L_{2}}\left(X\right)-c_{L_{1}}\left(X\right)\right)\times \hat{d}\left(U\right),\\ d_{\Pi }\left(X\right) & =-n_{\Pi }^{⊤}\left(X\right)c_{L_{1}}\left(X\right), \end{array}\right.$$

where $c_{L_{j}}$ denotes a point on the *j*-th visible edge and $\hat{d}$ represents the axis direction. The plane Π is defined by the form $n_{\Pi }^{⊤}x+d_{\Pi }=0$, where $n_{\Pi }\in R^{3}$ is the plane normal and $x\in R^{3}$ denotes a point on Π. By intersecting the viewing ray $v_{l_{j}}^{k}$ with the plane Π, the back-projected 3D point $q_{L_{j}}^{k}$ is obtained as

(22) $$q_{L_{j}}^{k}\left(X\right)=-\frac{d_{\Pi }\left(X\right)}{n_{\Pi }^{⊤}\left(X\right)v_{l_{j}}^{k}}v_{l_{j}}^{k}.$$

The edge alignment error term associated with edge $L_{j}$ is defined as the distance from $q_{L_{j}}^{k}$ to $L_{j}$:

(23) $$e_{L_{j}}^{k}\left(X\right)=∥m_{L_{j}}\left(U,W\right)-q_{L_{j}}^{k}\left(X\right)\times {\hat{d}}_{L_{j}}\left(U\right)∥.$$

Finally, the total energy is formulated as the mean squared errors over all endpoints:

(24) $$E_{L}\left(X\right)=\frac{1}{4}\sum_{j=1}^{2}\sum_{k=1}^{2}{\left(e_{L_{j}}^{k}\left(X\right)\right)}^{2}.$$

### 4.4. Solver

The minimization of (15) is a weighted nonlinear least-squares problem, which is solved by using a coarse-to-fine strategy. Specifically, an initial estimate $X_{0}$ is first obtained via a RANSAC-based method [7] and subsequently refined through iterative optimization.

Since the variables (U,W) associated with the central axis must reside on manifolds SO(3)×SO(2) during iteration, we adopt the optimization technique on manifold [27]. At the *n*-th iteration, a perturbation vector $\delta ={\left(\delta \theta^{⊤},\delta \phi ,\delta r\right)}^{⊤}\in R^{5}$ is applied to the current estimate $X_{n}$, resulting in an updated state defined as

(25) $$X_{n}⊕\delta =\left\{U_{n}\mathrm{Exp}\left(\delta \theta \right),W_{n}\mathrm{Exp}\left(\delta \phi \right),r_{n}+\delta r\right\}.$$

By linearizing (15) around the current estimate $X_{n}$, the objective function (15) is approximated as

(26) $$\begin{array}{c} F\left(X_{n}⊕\delta \right)\approx {∥\Omega^{\frac{1}{2}}e_{n}+\Omega^{\frac{1}{2}}J_{n}\delta ∥}^{2}, \end{array}$$

where $e_{n}\in R^{N+4}$ is the stacked error vector and $J_{n}\in R^{(N+4)\times 5}$ is the Jacobian matrix:

(27) $$e_{n}=\left[\begin{array}{c} e_{1}\left(X_{n}\right)\\ \vdots \\ e_{N}\left(X_{n}\right)\\ e_{L_{1}}^{1}\left(X_{n}\right)\\ e_{L_{1}}^{2}\left(X_{n}\right)\\ e_{L_{2}}^{1}\left(X_{n}\right)\\ e_{L_{2}}^{2}\left(X_{n}\right) \end{array}\right],\;J_{n}=\left[\begin{array}{c} {\left.\frac{\partial e_{1}\left(X_{n}⊕\delta \right)}{\partial \delta }\right|}_{\delta =0}\\ \vdots \\ {\left.\frac{\partial e_{N}\left(X_{n}⊕\delta \right)}{\partial \delta }\right|}_{\delta =0}\\ {\left.\frac{\partial e_{L_{1}}^{1}\left(X_{n}⊕\delta \right)}{\partial \delta }\right|}_{\delta =0}\\ {\left.\frac{\partial e_{L_{1}}^{2}\left(X_{n}⊕\delta \right)}{\partial \delta }\right|}_{\delta =0}\\ {\left.\frac{\partial e_{L_{2}}^{1}\left(X_{n}⊕\delta \right)}{\partial \delta }\right|}_{\delta =0}\\ {\left.\frac{\partial e_{L_{2}}^{2}\left(X_{n}⊕\delta \right)}{\partial \delta }\right|}_{\delta =0} \end{array}\right].$$

The weighting matrix Ω balances the contributions of the point-to-cylinder term and the edge alignment term in the overall objective function, and is defined as

(28) $$\Omega =\left[\begin{array}{cc} \frac{1-w_{L}}{N}I_{N} & 0_{N\times 4}\\ 0_{4\times N} & \frac{w_{L}}{4}I_{4} \end{array}\right].$$

To minimize the formulated weighted nonlinear least squares problem, we adopt the Levenberg–Marquardt (LM) algorithm [28]. At each iteration, the update step $\delta^{*}$ is computed by solving the following normal equations:

(29) $$\left(J_{n}^{⊤}\Omega J_{n}+\lambda I_{5}\right)\delta^{*}=-J_{n}^{⊤}\Omega e_{n}.$$

where λ denotes the damping factor. If the update reduces the cost $F(X_{n}⊕\delta^{*})$, then the update step $X_{n}\leftarrow X_{n}⊕\delta^{*}$ is accepted and λ is decreased; otherwise, the update is rejected and λ is increased. The complete procedure is summarized in Algorithm 2.
**Algorithm 2:** Iterative Cylinder Refinement via Edge-Point Cloud Fusion*Initialization.*The initial guess $X_{0}$,the point set $P={\left\{p_{i}\in R^{3}\right\}}_{i=1}^{N}$,the observed edge pairs $Q_{\mathrm{obs}}=\left\{O_{1},O_{2}\right\}$,damping factor $\lambda =10^{-3}$, and scale factor ν=10.Set the current estimate $X_{n}\leftarrow X_{0}$.Construct the weighting matrix Ω via Equation (28).*Update.* (Optimization Loop)Under $X_{n}$, extract the associated edge set Q←AssociateEdges ($X_{n},Q_{\mathrm{obs}}$).Construct the stacked error vector $e_{n}$ and the Jacobian matrix $J_{n}$ via Equation (27).Compute the update step $\delta^{*}$ via Equation (29).If $F\left(X_{n}⊕\delta^{*}\right)<F\left(X_{n}\right)$, accept the update $X_{n}\leftarrow X_{n}⊕\delta^{*}$ and decrease λ←λ/ν.Otherwise, reject the update and increase λ←λ·ν.*Termination.*Repeat the *Update* step until convergence.Output the optimized parameters $X^{*}=X_{n}$.

### 4.5. Applications

The optimized cylinder parameters $X^{*}$ serve as a geometric prior for downstream tasks. This section presents two downstream applications enabled by the estimated cylinder model: model-based point cloud completion and finite extent recovery for cylindrical objects.

#### 4.5.1. Model-Based Point Cloud Completion

Due to the sensor’s inherent limitations and measurement noise, raw point cloud data P often suffer from missing or corrupted regions. To address this, we employ a ray tracing-based approach [29] to restore surface geometry. Based on the optimized cylinder parameters $X^{*}$, points in the masked region M are reconstructed by intersecting the cylinder model with rays originating from the camera optical center o. This process yields geometrically complete point cloud data $P^{*}$.

#### 4.5.2. Finite Extent Recovery

Although the optimized parameter set $X^{*}$ corresponds to an infinite cylinder, real-world applications require a finite-length cylinder representation. Since single-view observations are not always complete, our objective is to recover the observed centroid $c_{0}$ and the observed length $l_{c}$ defined on the masked region M of the cylindrical surface. Specifically, we define $l_{c}$ as the maximal extent of the visible cylindrical surface and set $c_{0}$ to be the midpoint of this extent. We first project the points in $P^{*}$ onto the estimated cylinder central axis, resulting in a projected point set $P_{⊥}^{*}$. The two endpoints $c_{e_{1}}$ and $c_{e_{2}}$ are obtained by maximizing the pairwise distance within the set $P_{⊥}^{*}$:

(30) $$\left\{c_{e_{1}},c_{e_{2}}\right\}=\arg\max_{\left\{c_{i},c_{j}\right\}\subset P_{⊥}^{*}}∥c_{i}-c_{j}∥.$$

The centroid of a finite cylinder $c_{0}$ is computed by the midpoint of the two endpoints, and the visible length $l_{c}$ is given by their distance:

(31) $$\left\{\begin{array}{cc} c_{0} & =\frac{1}{2}\left(c_{e_{1}}+c_{e_{2}}\right),\\ l_{c} & =∥c_{e_{1}}-c_{e_{2}}∥. \end{array}\right.$$

This formulation is independent of prior assumptions on object length and accommodates both full and partial visibility. When the observed surface covers the full span of the cylindrical object, the estimated parameters align with the physical centroid and length. Under partial observability (e.g., due to invisible end-faces or external occlusion), the method recovers the centroid and axial extent of the visible segment.

## 5. Experiments

In this section, we conduct a series of experiments to provide a comprehensive evaluation of the proposed edge–point cloud fusion method. All experiments are performed on a desktop with an Intel i5-12400F CPU (Intel Corporation, Santa Clara, CA, USA, 32 GB RAM, and an NVIDIA RTX 3060Ti GPU (NVIDIA Corporation, Santa Clara, CA, USA). We implement our method in JAX [30] for numerical computing, which enables GPU hardware to accelerate the computations.

This section is organized as follows: Section 5.1 introduces the dataset and evaluation metrics; Section 5.2 describes the baseline methods; in Section 5.3, we conduct an ablation study on the edge fusion weight, while Section 5.4 performs the sensitivity analysis; Section 5.5 presents comparison results against other methods; Section 5.6 presents the computational efficiency analysis; finally, we provide an application in Section 5.7 by demonstrating its performance in a real-world piping environment.

### 5.1. Datasets and Evaluation Metrics

To conduct experiments under controlled conditions, we use a consumer-grade RGB-D camera (Astra+, Orbbec Technology Co., Ltd., Shenzhen, China) for data acquisition, which measures depth data by a monocular speckled structured-light technique. To improve pixel-level alignment, we perform camera calibration using the standard checkerboard-based method [31], as detailed in [15]. The raw depth images are subsequently registered to the RGB coordinate system to generate the aligned RGB-D input required by our method. The image resolution is set to 640×480 pixels. Standard aluminum cylinders with radii $r_{g}\in \left\{20,30,40,50,60\right\}$ mm and a fixed length of 100 mm are considered as testing targets. This range of radii is selected in order to assess performance across different cylinder sizes.

#### 5.1.1. Data Acquisition with Viewpoint Variations

**Figure 4:**
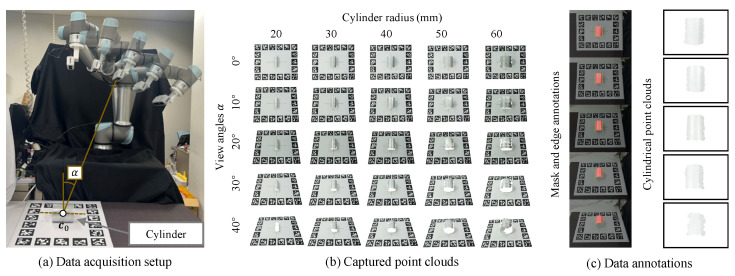
Experimental setup and data preparation. The RGB-D data acquisition process with (**a**) viewpoint variations, (**b**) example point cloud data under different viewpoints, and (**c**) data annotations, including mask M, edge set $Q_{\mathrm{obs}}$, and observed point cloud data P.

To assess the performance of the proposed method under different observation conditions, we introduce variations in the camera viewpoint. A six-degree-of-freedom industrial robot arm (UR5e, Universal Robots, Odense, Denmark) is used to control the camera motion. As shown in Figure 4a, an RGB-D camera is mounted on the end-effector of the robot arm, allowing the camera to be moved to a target pose under consistent and reproducible motion conditions. All data were collected under stable indoor lighting conditions.

**Figure 5:**
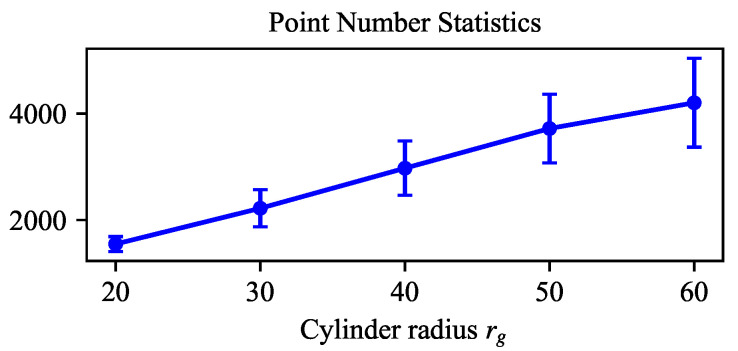
Point number statistics under different cylinder radii $r_{g}$. The marker point indicates the mean point number over tilt angles α and the error bars denote standard deviation (Std) values.

The target cylinder is placed at the center of a planar board with fiducial AprilTag markers [32]. The board provides visual references for guiding the robot during camera motion and enables the establishment of ground-truth references. We define the camera viewpoint by the tilt angle α and the working distance $d_{w}$, with the target cylinder appearing at the center of the image, as shown in Figure 4a. Within the camera’s operating range, the tilt angle is specified as α∈{0°,10°,20°,30°,40°} and the working distance is fixed at $d_{w}=0.7$ m. Figure 4b shows an example of the captured point cloud data. In the extreme case of large tilt angles and small radii (i.e., α=40° and $r_{g}=20$ mm), data acquisition fails due to the sensor’s inherent limitations when measuring highly curved surfaces at oblique viewing angles. This setup yields 24 valid configurations, with the robot remaining stationary for each one while capturing 20 consecutive RGB-D images in order to evaluate repeatability. Figure 5 reports the point number statistics of different cylinder radii $r_{g}$ in the dataset.

To establish ground-truth references, we follow the procedures from Hinterstoisser et al. [33]. The target cylinder is manually placed at the center of the planar board so that the center point and axis direction relative to the planar board are known. The camera pose relative to the planar board is computed by solving the Perspective-n-Point (PnP) problem using the method in [34]. Therefore, the complete ground-truth cylinder parameters in the camera frame are determined by combining the known radius and length with the estimated center point and axis direction.

#### 5.1.2. Data Annotation

We employ manual annotations for the cylindrical region M and edge segments $Q_{\mathrm{obs}}$ to ensure a strictly controlled evaluation. Specifically, the cylindrical region M in the RGB images is annotated using the Labelme tool [35] and the point cloud data P belonging to the target cylinder are extracted by back-projecting the depth images. The two visible longitudinal edges are manually labeled to generate the observed edge set $Q_{\mathrm{obs}}$, which lies along the object boundaries in the RGB images. Figure 4c illustrates an example of the annotated data. This experimental design explicitly decouples geometric parameter estimation errors from potential uncertainties introduced by upstream detection modules. This isolation allows for a rigorous benchmarking of the theoretical upper bound on the accuracy and robustness of the proposed method.

#### 5.1.3. Evaluation Metrics

We evaluate estimation accuracy using four metrics: orientation error $E_{o}$, position error $E_{p}$, relative radius error $E_{r}$, and relative length error $E_{l}$. Let the estimated parameters be $\left\{c_{0,e},{\hat{d}}_{e},r_{e},l_{c,e}\right\}$ and the ground truth $\left\{c_{0,g},{\hat{d}}_{g},r_{g},l_{c,g}\right\}$. The metrics are defined as

(32) $$\left\{\begin{array}{c} E_{o}=\cos^{-1}∥{\hat{d}}_{e}^{⊤}{\hat{d}}_{g}∥,\\ E_{p}=∥c_{0,e}-c_{0,g}∥,\\ E_{r}=\frac{∥r_{e}-r_{g}∥}{r_{g}}\cdot 100\%,\\ E_{l}=\frac{∥l_{c,e}-l_{c,g}∥}{l_{c,g}}\cdot 100\%. \end{array}\right.$$

### 5.2. Baseline Methods

The proposed method is evaluated against three representative point-based cylinder fitting baselines: a RANSAC-based approach [7] as implemented in [36], and two least squares-based methods proposed by Eberly [37] and Zhang et al. [15]. Since the proposed method operates as a backend refinement module, RANSAC is used both to initialize the solver and as a baseline representing the coarse initial estimate. Eberly’s method [37] estimates the cylinder’s orientation by minimizing a quadratic form, followed by closed-form solutions of the remaining geometric parameters. The method of Zhang et al. [15] is a special case of the proposed method when the edge fusion weight $w_{L}$ is set to zero. All methods follow their default implementation. To ensure a fair comparison, both least squares-based baselines are initialized with the same RANSAC-based estimate as ours.

It is worth noting that the three baseline methods rely solely on point information. Furthermore, these methods estimate an infinite cylinder model, and as such do not directly provide the object centroid or finite length. To ensure a consistent comparison, we apply the same postprocessing procedure described in Section 4.5 to recover these parameters for all compared methods.

### 5.3. Ablation Study on Edge Fusion Weight

To analyze the effect of the edge-fusion weight $w_{L}$, we use a small validation subset consisting of the first frame from six representative configurations of the complete dataset. These configurations are defined by three cylinder radii $r_{g}\in \left\{20,30,60\right\}$ mm and two tilt angles α∈{0°,30°}. A grid search over $w_{L}\in \left\{0,0.1,\ldots ,1.0\right\}$ is then performed on this validation subset.

**Figure 6:**
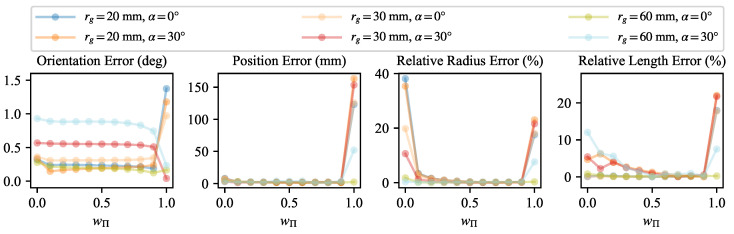
Effect of the edge-fusion weight $w_{L}\in \left\{0,0.1,\ldots ,1.0\right\}$ across radii ($r_{g}\in \left\{20,30,60\right\}$ mm) and tilt angles (α∈{0°,30°}).

As shown in Figure 6, when $w_{L}=0$ (i.e., using point term only), performance degrades for $r_{g}=20$ mm cylinders, while $r_{g}=60$ mm cylinders are less affected. This is due to the inherent limitations of the RGB-D sensor when measuring surfaces with high curvature, which make the point cloud data unreliable and degrade the parameter estimation performance. When $w_{L}=1$ (edge term only), the solver becomes unstable, as the back-projection-based edge alignment leads to an ill-conditioned optimization problem. In the absence of point-based metric constraints, the cylinder scale becomes weakly constrained, especially the radius, resulting in ambiguous or unreliable parameter updates. As a result, the radius is not reliably optimized and tends to remain close to its initial value, while only the central axis is refined. In contrast, the weight values ($w_{L}\in \left[0.5,0.8\right]$) lead to improved accuracy across all evaluated configurations. This result highlights the roles of the two energy terms in parameter estimation. The absolute metric scale is provided by the point-to-cylinder term $E_{P}$, and geometric consistency is additionally enforced by the edge alignment term $E_{L}$. Based on this analysis, we choose $w_{L}=0.6$ for all subsequent experiments, since this value provides consistent performance across different configurations.

### 5.4. Sensitivity Analysis

This section presents a sensitivity analysis of the proposed method. We evaluate the robustness of the proposed method against perturbations in the edge observations as well as in the solver’s initialization, both of which are critical factors. The analysis is conducted on the validation subset defined in Section 5.3.

#### 5.4.1. Robustness to Edge Perturbation

**Figure 7:**
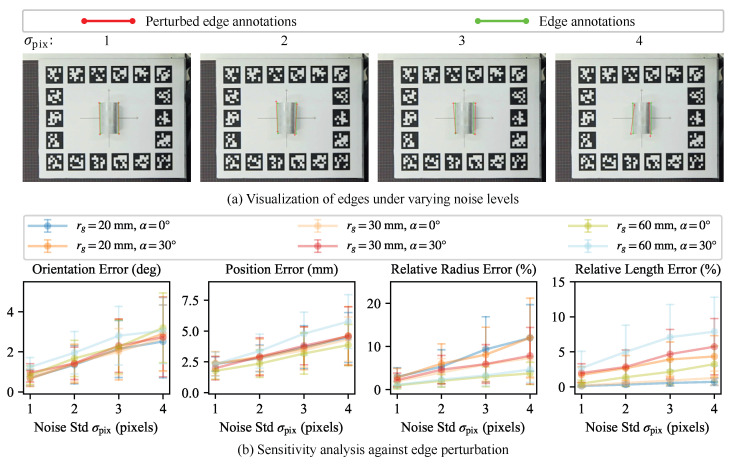
Sensitivity analysis for edge perturbation. (**a**) Visualization of synthetic edge noise generated with $r_{g}=30$ mm and α=0° for different $\sigma_{\mathrm{pix}}$ values. Red and green lines represent perturbed ($\sigma_{\mathrm{pix}}\in \left\{1,2,3,4\right\}$ pixels) and original annotations, respectively. (**b**) Impact of perturbed edge on parameter estimation accuracy. The points indicate the mean error and error bars denoting the Std.

To simulate noisy edge observations, we introduce synthetic perturbations to the endpoint coordinates of the observed edge set $Q_{\mathrm{obs}}$. Specifically, each endpoint $q_{m}^{k}\in Q_{\mathrm{obs}}$ is corrupted by additive zero-mean isotropic Gaussian noise. To control the noise level, the standard deviation (Std) value of the noise is specified as $\sigma_{\mathrm{pix}}\in \left\{1,2,3,4\right\}$ pixels. As shown in Figure 7a, larger $\sigma_{\mathrm{pix}}$ values will lead to more significant deviations from the original edge annotations. For each level, 100 independent trials were performed.

Figure 7b presents the statistical results of the proposed method under different levels of edge perturbation. As the perturbation level increases, our method exhibits a gradual degradation in estimation accuracy. At high perturbation levels ($\sigma_{\mathrm{pix}}=4$ pixels), the mean values of $E_{o}$, $E_{p}$, $E_{r}$, and $E_{l}$ reach up to 3.20°, 5.77 mm, 12.02%, and 7.89%, respectively. The Std values also exhibit relatively high magnitudes, indicating increased estimation variability. This performance degradation can be attributed to the inherent uncertainty in point cloud data, which is further amplified by severe corruption of edge observations. Nevertheless, the proposed method demonstrates robust performance under moderate edge perturbations ($\sigma_{\mathrm{pix}}\leq 2$ pixels). In this situation, the mean values of $E_{o}$, $E_{p}$, $E_{r}$, and $E_{l}$ remain below 1.96°, 3.38 mm, 6.00%, and 5.00%, respectively, with relatively small Std values. These results indicate that the proposed method is tolerant to inaccurate edge observations and can be integrated into practical perception pipelines under reasonable noise conditions.

#### 5.4.2. Robustness to Initialization Perturbations

To evaluate the robustness of the proposed iterative solver to initialization perturbations, we perform a quantitative sensitivity analysis. The edge alignment energy term depends on a back-projection plane Π computed from the cylinder parameters X. Therefore, large deviations in the initial estimate $X_{0}$ may distort the geometry of Π, weakening the geometric consistency of the edge alignment constraints during iterative optimization.

**Figure 8:**
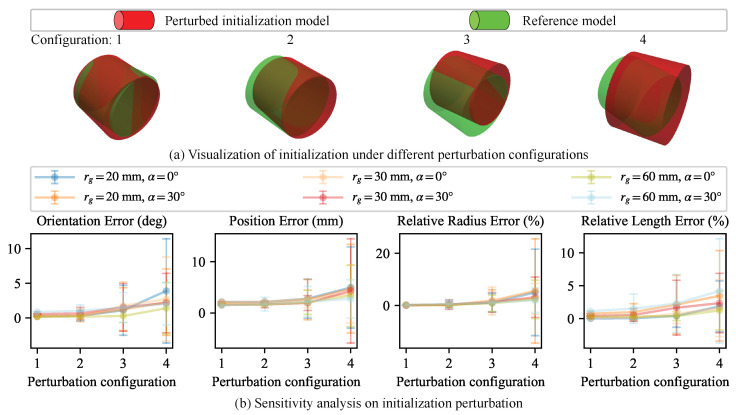
Sensitivity analysis for initialization perturbation. (**a**) Visualization of the perturbed initial estimates (red) versus the ground-truth reference (green). The misalignment becomes visibly severe as the perturbation severity increases. (**b**) Parameter estimation accuracy under different configurations defined in Table 1. The markers indicate the mean error, and the error bars denote the Std.

To assess the solver under such conditions, we perturb the ground-truth cylinder parameters with zero-mean isotropic Gaussian noise to generate perturbed initial estimates. Specifically, we apply perturbations to the ground-truth direction ${\hat{d}}_{g}$, center point $c_{0,g}$, and radius $r_{g}$:

(33) $$\left\{\begin{array}{cc} {\hat{d}}_{0}^{n}=\mathrm{Exp}\left(n_{d}\right)\;{\hat{d}}_{g}, & n_{d}∼N\left(0,\sigma_{d}^{2}I_{3}\right),\\ c_{0}^{n}=c_{0,g}+n_{c},\; & n_{c}∼N\left(0,\sigma_{p}^{2}I_{3}\right),\\ r_{0}^{n}=r_{g}+n_{r},\; & n_{r}∼N\left(0,{\left(\sigma_{r}r_{g}\right)}^{2}\right), \end{array}\right.$$

where the unit-length constraint $∥{\hat{d}}_{0}^{n}∥=1$ is preserved because $\mathrm{Exp}\left(n_{d}\right)\in \mathrm{SO}\left(3\right)$ is an orthogonal matrix. Here, the parameters $\sigma_{d}$, $\sigma_{p}$, and $\sigma_{r}$ control the perturbation magnitudes of direction, position, and relative radius, respectively. We consider three severity levels and evaluate four noise configurations (one low, two medium, and one high), as summarized in Table 1. For each configuration, we perform 100 independent trials. Figure 8a visualizes the perturbed initial estimates under different severity levels.

Figure 8b reports the quantitative results. The proposed method remains robust under low and medium perturbations, consistently refining the perturbed initial estimates with stable convergence. When the perturbation reaches a high level, the performance degrades, indicating that highly inaccurate initializations can compromise the informativeness of the edge alignment constraints. In practice, the RANSAC-based initialization in our pipeline provides sufficiently accurate initial estimates, so the solver typically converges to a consistent solution.

### 5.5. Comparison with Baseline Methods

#### 5.5.1. Quantitative Comparison

**Figure 9:**
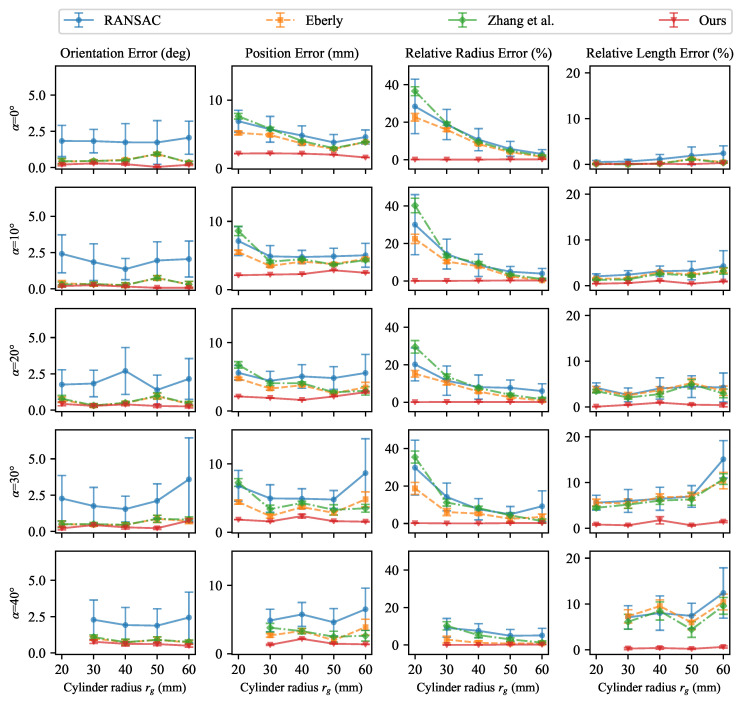
Quantitative comparison results across all configurations. The plots show the mean as the point and the Std as error bars.

Figure 9 presents quantitative comparison results obtained from repeated RGB-D captures. For each metric, the values of mean and Std are reported. Our method achieves mean errors below 0.77° for $E_{o}$, 2.74 mm for $E_{p}$, 0.24% for $E_{r}$, and 1.76% for $E_{l}$ across all settings, while also exhibiting the smallest Std over repeated runs. In contrast, point-based baseline methods are sensitive to data acquisition conditions, particularly variations in the cylinder radius $r_{g}$ and tilt angle α. RANSAC shows pronounced instability because it serves as the initialization step, whereas the proposed edge–point fusion strategy effectively refines the rough initial estimate and achieves accurate solutions despite inaccurate coarse initialization. However, the methods of Eberly [37] and Zhang et al. [15], both of which rely solely on point cloud data, show limited parameter estimation accuracy even when initialized with the same RANSAC-based estimate. In particular, reducing the cylinder radius $r_{g}$ leads to marked degradation in estimation accuracy. In addition, variations in the tilt angle α further result in noticeable changes in both the mean errors and Std values, with the impact being especially evident for $E_{p}$ and $E_{l}$ at larger tilt angles α. Because the cylinder centroid and length are recovered using a model-based approach, errors in the upstream parameter estimation tend to accumulate and become amplified, an effect that is more pronounced at higher tilt angles. As the curvature of the cylindrical surface increases or the tilt angle α becomes larger, the employed RGB-D camera struggles to capture accurate point cloud information, which directly leads to a performance degradation in such point-based methods.

These results show that the proposed method remains reliable and robust under different cylinder radius $r_{g}$ and the tilt angle α. Although the quality of point cloud data is affected for small-radius cylinders and large tilt angles, the integration of edge information effectively compensates for the limitations of point data, leading to improved estimation accuracy and robustness. In addition, the edge fusion weight value $w_{L}=0.6$ determined from a validation subset remains effective beyond the validation settings and can be generalized in the complete dataset.

#### 5.5.2. Qualitative Comparison

**Figure 10:**
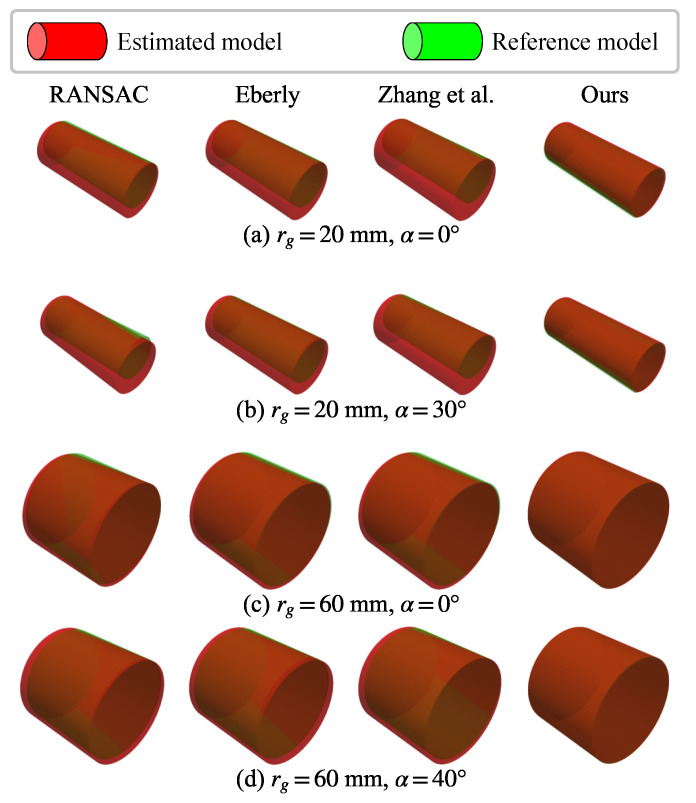
Visualization of the estimated cylinder model and the reference model on representative examples. Our method aligns better with the reference than the point-only baselines.

A comparison between the estimated cylinder and the ground-truth reference model for representative cases is shown in Figure 10. These results show that RANSAC exhibits a noticeable deviation in orientation, whereas the methods proposed by Eberly [37] and Zhang et al. [15] achieve similar orientation estimation. Although all baseline methods demonstrate strong performance for the large-radius target ($r_{g}=60$ mm), they tend to overestimate the radius and exhibit position drift in the case of the small-radius target ($r_{g}=20$ mm). By contrast, our approach produces cylinder estimates with an alignment that is more consistent with the ground truth.

**Figure 11:**
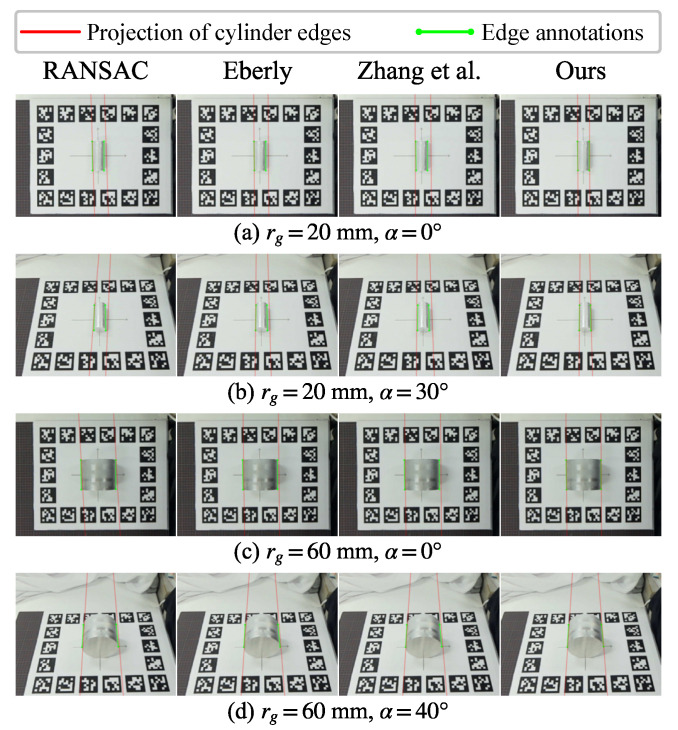
Projected cylinder edges overlaid on the annotated image edges in representative examples. Our method aligns the observed longitudinal edges, whereas the point-only baselines exhibit noticeable drift.

Figure 11 shows the visualization of projected edges. The projection that is closer to the annotation suggests that the estimated cylinder parameters better satisfy the projection relationship. As in the visualization of cylinder model comparison, the baseline methods show significant offsets from the annotated edges. By explicitly considering the edge information, our method achieves better alignment, further verifying the accuracy of the estimated parameters.

**Figure 12:**
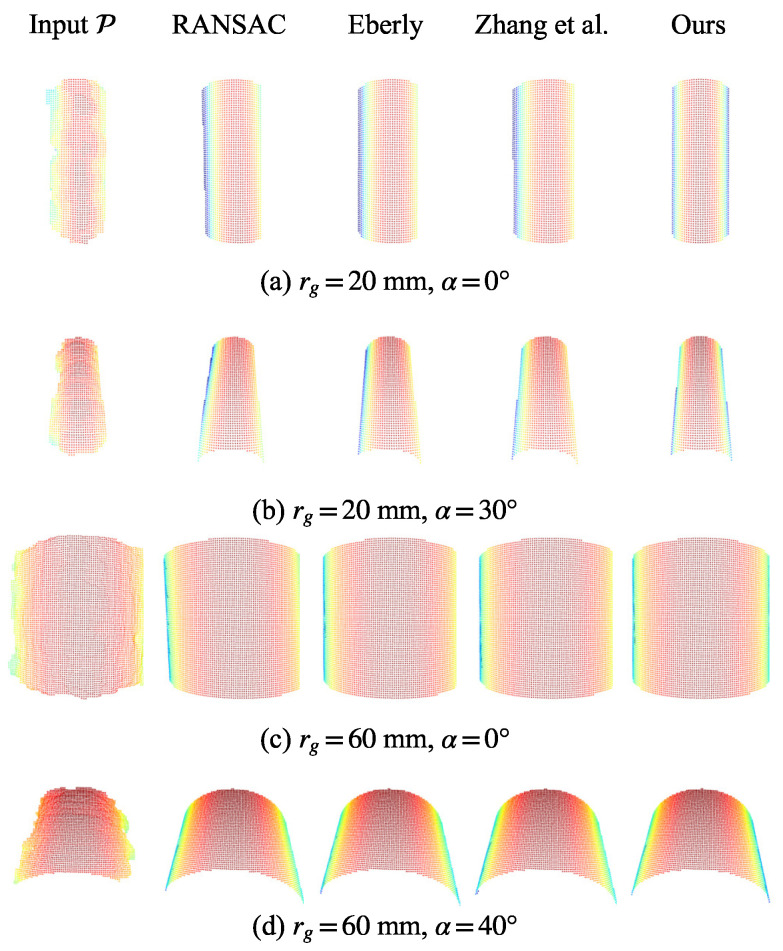
Point cloud completion results. Our method fills the missing parts, without the holes or shape distortions seen in the baselines.

Figure 12 presents a qualitative comparison of the point cloud completion results. As an intermediate result, accurate parameter estimation will produce higher-quality point cloud completion. The baseline methods produce incomplete reconstructions with noticeable geometric distortions, primarily due to inaccurate parameter estimation. In contrast, the proposed method yields a geometrically consistent completion of the cylindrical point cloud. Owing to this geometric consistency, the cylinder length and centroid can be reliably recovered using the model-based approach, resulting in more accurate estimates.

### 5.6. Computation Efficiency Analysis

Table 2 presents the running time of each method, where we report the average and Std over all the datasets used in our experiments. Notably, RANSAC and Eberly’s method are implemented on the CPU, whereas the method of Zhang et al. [15] and our approach are implemented on the GPU.

RANSAC is the fastest method and achieves the shortest runtime of 0.017 s, as it is used for coarse initialization and obtains an approximate solution through random sampling. By contrast, Eberly’s method is the slowest, requiring 7.904 s and exhibiting a large Std of 2.715 s. This is because the method is implemented on the CPU, and as such is sensitive to changes in the size of the input point clouds. Denser point clouds lead to higher computational cost, while variations in point cloud size result in less stable runtimes.

To ensure a fair comparison, we evaluate our method against the method of Zhang et al. [15] under the same hardware settings. Both methods benefit from CPU and GPU acceleration and achieve good runtimes. While the method of Zhang et al. [15] optimizes only the point-based term $E_{p}$, it achieves an average runtime of 0.565 s. In contrast, our method attains an average runtime of 0.805 s. The 0.240 s time overhead results from the extra computation introduced by the edge alignment constraints in the fusion strategy. Although this causes a moderate overhead, the computational efficiency remains high owing to the large gains in accuracy and robustness.

### 5.7. Application Demonstration on a Real-World Piping Environment

**Figure 13:**
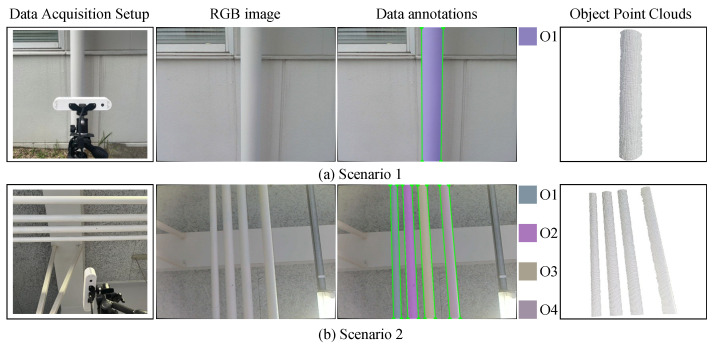
Data acquisition setup and annotation results in real-world piping scenarios. Scenario S2-O1 has a radius of $r_{g}=19$ mm, while Scenarios S2-O2 through S2-O4 each have a radius of $r_{g}=25.5$ mm.

To illustrate the practical applicability of the proposed method under real-world sensing conditions, we present a field demonstration in a piping scenario. As shown in Figure 13, a tripod-mounted RGB-D camera is oriented to face a number of straight pipes for data acquisition. Scenario 1 (S1) consists of an outdoor wall-mounted pipe (S1-O1) with a radius of $r_{g}=57$ mm. Scenario 2 (S2) consists of an indoor ceiling-mounted piping system composed of four smaller pipes: S2-O1 has a radius of $r_{g}=19$ mm, while S2-O2 through S2-O4 each have a radius of $r_{g}=25.5$ mm. Since real-world piping environments make it difficult to establish reliable ground truth for complete cylinder parameters, this subsection focuses on an application-oriented demonstration rather than a strict quantitative evaluation. For pipes with known radii $r_{g}$, we report radius estimation results as an indicative quantitative metric. To facilitate qualitative comparison under real sensing conditions, we also provide visualizations of edge reprojection and reconstructed piping models.

**Figure 14:**
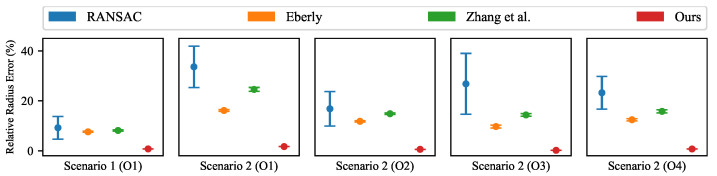
Comparison of relative radius error in real-world piping scenarios over repeated captures.

**Figure 15:**
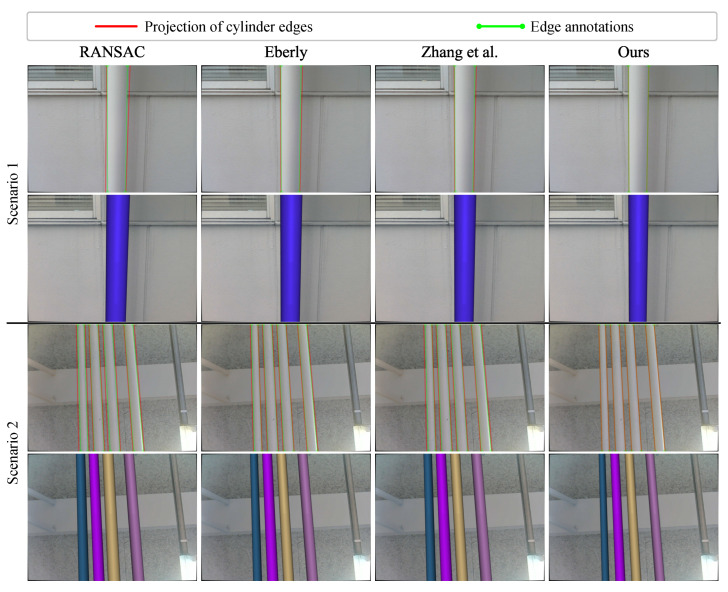
Visual comparison using real-world piping data. The point-based baselines show visible drift, while our method more accurately aligns the piping boundaries in the RGB images.

Figure 14 presents the quantitative results of radius estimation. Our method achieves lower mean values of $E_{r}$ and keeps smaller Std values across all tested pipes. This result shows higher accuracy and better robustness when estimating pipe radii in real-world conditions. In contrast, the three point-based baseline methods produce much larger errors in radius estimation. Figure 15 shows the differences between these methods through edge projections. Since our method uses edge information, the projected edges closely follow the annotated piping boundaries. This indicates that the central axis (i.e., the orientation and position) of the pipes is well-estimated. On the other hand, the baseline methods show clear drift in their edge projections. This drift indicates inaccurate parameter estimation in the central axis. As a result, the reconstructed piping models from the baseline methods do not match the true piping geometry. By comparison, our method produces geometrically consistent piping models. Notably, although the end-faces of the pipes are not fully visible, the finite extents are recovered to represent the observed pipe segments. This consistency is shown by the better alignment with the observed piping boundaries in the RGB images.

## 6. Discussion

This section discusses the proposed method, its limitations, and directions for future research.

### 6.1. Limitations

Although the proposed edge–point fusion method achieves consistent improvements under the tested conditions, real-world performance will depend on uncertainties introduced by upstream perception modules. In practical deployments, cylindrical regions and edge features are typically produced by automatic detection, segmentation, and edge extraction pipelines, which can be sensitive to scene factors such as lighting variation and background texture. To evaluate the fusion-based geometric fitting itself in a controlled setting, we use manual annotations in this study. This choice isolates the proposed formulation from detector-dependent errors, but also means that upstream failures (e.g., missing, biased, or spurious edges) are not explicitly modeled. Within an end-to-end perception pipeline, the fusion-based fitting component would be applied downstream of standard perception modules. While modeling detector-induced uncertainty and validating the full pipeline are beyond the scope of this geometry-centric study, they remain important directions for future work, as they require jointly considering both upstream perception and downstream fitting.

In our experiments, we use a fixed edge fusion weight that is selected on a validation subset and then applied to all evaluated configurations. Despite its effectiveness being validated under the tested conditions, a fixed weighting can be suboptimal across different RGB-D sensors and diverse scenes. For example, a fixed setting may fail to adapt to the changing reliability of the two modalities in low-texture scenes where edge confidence is reduced or in the presence of severe point cloud noise and outliers. Moreover, while we evaluate different radii and viewing angles, extreme conditions such as highly specular reflections or heavy occlusion were not explicitly modeled. These limitations motivate adaptive fusion strategies guided by confidence and noise characteristics as well as more explicit robustness to outliers.

Finally, the current work assumes ideal cylindrical geometry. As such, its direct applicability is limited for objects often encountered in real-world settings that deviate substantially from ideal cylinders, such as hoses, cables, or deformed pipes. More flexible shape parameterizations would be beneficial in extending the proposed method beyond ideal cylinders.

### 6.2. Future Perspectives

The results suggest that the proposed method improves robustness under challenging conditions such as small cylinder radii and large viewing angles, which commonly occur with consumer-grade RGB-D sensors. Given that this study focuses on fusion-based geometric fitting under controlled settings, several directions remain to improve practical deployment and broaden applicability. (1) A natural extension would be to integrate an automatic detector in order to realize a fully end-to-end system in which detection, edge extraction, and parameter estimation are performed within a single pipeline without manual annotation. To reduce reliance on manual annotation, future work could study self-supervised or weakly supervised schemes that leverage temporal or multi-view consistency constraints to support automatic annotation generation [38]. (2) An adaptive weighting strategy could be explored to dynamically balance the contributions of point and edge constraints according to sensor characteristics and scene conditions. For instance, the method could incorporate sensor noise models or edge confidence maps to adaptively reduce edge weight in low-texture environments, and the fusion weights could be adjusted according to point measurement noise and sampling density. (3) Beyond ideal cylindrical shapes, the current method can be extended to support approximately cylindrical objects. One possible direction is to generalize the central axis representation as a smooth curve with spatially varying radius. Under such a formulation, edge information may still provide useful geometric constraints to compensate for point cloud inaccuracies on curved or deformable surfaces, broadening applicability to real-world perception tasks. (4) In addition to detector integration, recent learning-based methods may complement the proposed geometric fitting method by providing candidate regions or initial hypotheses that reduce the downstream search space [39] and by providing image-conditioned priors for point cloud denoising and completion under partial or noisy observations [40]. Integrating such components with the proposed formulation is promising, but requires careful treatment of uncertainty propagation and systematic error sources.

## 7. Conclusions

This paper proposes an edge–point cloud fusion approach for estimating cylinder parameters. By leveraging edge features as an additional geometric source, the proposed method jointly optimizes the full set of cylinder parameters by fusing edge-derived constraints with point cloud information. The experiments show significant improvements in accuracy and robustness for the proposed method compared with point-based fitting approaches. Future work will focus on achieving fully automatic processing and extending the method to handle approximately cylindrical structures and more complex geometric shapes.

## Figures and Tables

**Table 1:** Initialization perturbation settings for the sensitivity analysis, showing the four perturbation configurations grouped into three severity levels.

Perturbation Severity	Configuration	$\sigma_{d}$ (Deg)	$\sigma_{p}$ (mm)	$\sigma_{r}$ (%)
Low	1	3	3	5
Medium	2	5	5	10
	3	8	8	15
High	4	10	15	20

**Table 2:** Computation time statistics. The results are reported as mean ± Std (in seconds) calculated over all valid configurations. Methods are grouped according to their hardware implementation. The reported runtimes are influenced by both algorithmic design and implementation details.

CPU Implementation		GPU Implementation	
RANSAC	Eberly	Zhang et al.	Ours
0.017±0.010	7.904±2.715	0.565±0.037	0.805±0.109

## Data Availability

The data presented in this study are available on request from the corresponding author.
